# Messenger RNA-encoded antibody approach for targeting extracellular and intracellular tau

**DOI:** 10.1093/braincomms/fcae100

**Published:** 2024-03-25

**Authors:** Patricia Wongsodirdjo, Alayna C Caruso, Alicia K Yong, Madeleine A Lester, Laura J Vella, Ya Hui Hung, Rebecca M Nisbet

**Affiliations:** The Florey Institute, Parkville, Victoria 3052, Australia; Florey Department of Neuroscience and Mental Health, The University of Melbourne, Parkville, Victoria 3052, Australia; The Florey Institute, Parkville, Victoria 3052, Australia; Florey Department of Neuroscience and Mental Health, The University of Melbourne, Parkville, Victoria 3052, Australia; The Florey Institute, Parkville, Victoria 3052, Australia; The Florey Institute, Parkville, Victoria 3052, Australia; Florey Department of Neuroscience and Mental Health, The University of Melbourne, Parkville, Victoria 3052, Australia; The Florey Institute, Parkville, Victoria 3052, Australia; Department of Surgery, The Royal Melbourne Hospital, The University of Melbourne, Parkville, Victoria 3052, Australia; The Florey Institute, Parkville, Victoria 3052, Australia; Florey Department of Neuroscience and Mental Health, The University of Melbourne, Parkville, Victoria 3052, Australia; The Florey Institute, Parkville, Victoria 3052, Australia; Florey Department of Neuroscience and Mental Health, The University of Melbourne, Parkville, Victoria 3052, Australia

**Keywords:** mRNA, tau, antibody, immunotherapy, Alzheimer’s disease

## Abstract

Monoclonal antibodies have emerged as a leading therapeutic agent for the treatment of disease, including Alzheimer’s disease. In the last year, two anti-amyloid monoclonal antibodies, lecanemab and aducanumab, have been approved in the USA for the treatment of Alzheimer’s disease, whilst several tau-targeting monoclonal antibodies are currently in clinical trials. Such antibodies, however, are expensive and timely to produce and require frequent dosing regimens to ensure disease-modifying effects. Synthetic *in vitro*-transcribed messenger RNA encoding antibodies for endogenous protein expression holds the potential to overcome many of the limitations associated with protein antibody production. Here, we have generated synthetic *in vitro*-transcribed messenger RNA encoding a tau-specific antibody as a full-sized immunoglobulin and as a single-chain variable fragment. *In vitro* transfection of human neuroblastoma SH-SY5Y cells demonstrated the ability of the synthetic messenger RNA to be translated into a functional tau-specific antibody. Furthermore, we show that the translation of the tau-specific single-chain variable fragment as an intrabody results in the specific engagement of intracellular tau. This work highlights the utility of messenger RNA for the delivery of antibody therapeutics, including intrabodies, for the targeting of tau in Alzheimer’s disease and other tauopathies.

## Teaser

Wongsodirdjo *et al*. report that messenger RNA encoding a conventional tau antibody and an intrabody can be delivered to mammalian cells and translated into functional antibodies, with the translated intrabody capable of engaging intracellular tau. This work highlights the utility of messenger RNA-encoded antibody therapeutics targeting tau.

## Graphical Abstract

**Graphical Abstract:**
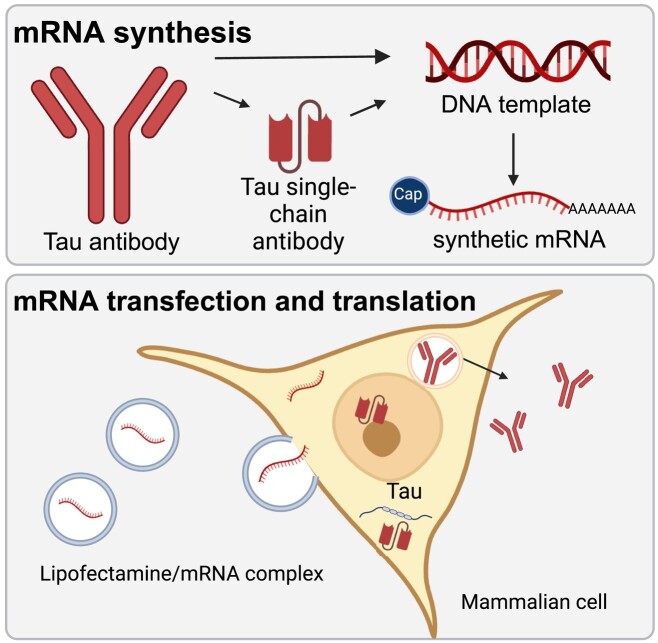

## Introduction

Alzheimer’s disease is the most common cause of dementia, a progressive neurodegenerative disease without a cure. One of the main pathological hallmarks of Alzheimer’s disease is the abnormal hyperphosphorylation and intraneuronal accumulation of the tau protein. Tau is a promising target for disease intervention, and pre-clinically, tau monoclonal antibodies have demonstrated an ability to reduce tau pathology in several tau transgenic mouse models.^1-6^ The ability of tau antibodies to improve disease outcomes in humans, however, has not yet been demonstrated, and many tau antibodies have been discontinued from clinical development.^7-10^ This is not surprising as conventional antibodies are unable to effectively cross the blood–brain barrier and enter the brain.^11,12^ Furthermore, there is limited evidence to suggest that conventional antibodies can transverse the neuronal cell membrane to engage intraneuronal tau.^13^ Conventional tau antibodies are, therefore, predicted to target extracellular tau and prevent tau pathology from spreading in a prion-like manner. The estimated pool of extracellular tau available for therapeutic targeting is only 0.001–0.01% of that of intracellular tau,^14,15^ however, suggesting that antibodies with enhanced targeting of intracellular tau may have better therapeutic outcomes.

Intracellular targeting of tau has previously been achieved in tau transgenic mouse models using intracellular antibodies (intrabodies).^16-18^ These intrabodies were generated by engineering the single-chain variable fragment (scFv) from the parental tau antibody and creating scFv-encoding adeno-associated viral vectors (AAVs). Intracerebral injection of the mice with the AAV resulted in neuronal transduction and the expressed tau intrabodies demonstrated successful reduction of pathogenic tau.^16-18^ Furthermore, comparison of an intrabody to a secreted scFv targeting tau revealed that targeting the intracellular population of tau has an enhanced therapeutic effect.^17^ Intrabody-mediated clearance of tau has also been achieved through the addition of functional peptides that direct the complex to the proteosome or induce autophagy.^16^ Whilst viral vector-mediated intrabody gene delivery is the current preferred strategy for intrabody delivery to the brain, clinical translation of AAV use is limited due to the presence of AAV neutralizing antibodies that are generated as a result of previous exposure to wild-type virus.^19^

A promising alternative to AAV-vectored antibodies is *in vitro*-transcribed (IVT) messenger RNA (mRNA). Synthetic IVT mRNA can be engineered at a fraction of the time and cost required to manufacture recombinant proteins and has fundamental advantages over viral-based systems, such as only requiring uptake into the cytosol, which results in immediate protein production. This makes mRNA a safe and fully controllable delivery tool and mRNA-encoded antibodies have recently been utilized pre-clinically for cancer and respiratory syncytial virus immunotherapy.^20^ Furthermore, IVT mRNA encoding single-domain antibodies specific for non-therapeutic targets were successfully expressed in living cells, facilitating intracellular protein targeting.^21^

Delivery of IVT mRNA encoding therapeutic antibodies and engineered intrabodies directly to neurons may transiently increase the amount of therapeutic protein in the brain compared with systemic protein delivery. We, therefore, wanted to determine the expression and tau engagement of a tau-specific antibody, RNJ1, in a conventional immunoglobulin (IgG) format and as engineered scFv intrabody following IVT mRNA delivery to human neuroblastoma SH-SY5Y cells. We demonstrate that transfection of SH-SY5Y cells with mRNA encoding the light chain (LC) and heavy chain (HC) of RNJ1 results in expression and successful generation of secreted RNJ1 IgG, capable of binding to human tau (hTau). Furthermore, transfection of SH-SY5Y cells with mRNA encoding the RNJ1 scFv results in RNJ1 intrabody expression and engagement of intracellular tau. Together, these findings validate mRNA as an important tool for Alzheimer’s disease therapeutic development.

## Materials and method

### Antibodies

Antibodies are as follows: mouse anti-green fluorescent protein (GFP) (Thermo Fisher Scientific; product number MA5-15256; immunoprecipitation: 1:200); mouse anti-β-actin [Merck; product number A2228; western blot (WB): 1:10 000]; rabbit anti-Flag (Cell Signalling Technology; product number 14793S; WB: 1:1000; immunofluorescence: 1:400); mouse anti-tau, RNJ1 (in-house; WB: 1:1000); mouse anti-Tau, Tau-5 (Merck; product number MAB361; specificity: bovine, human, murine tau; WB: 1:1000; immunofluorescence: 1:500; immunoprecipitation: 1:1000); anti-tau, HJ8.5 (in-house; WB: 1:1000); goat anti-mouse IRdye800CW (LI-COR; product number 926-32210; WB: 1:10 000); goat anti-rabbit IRdye680 (LI-COR; product number 926-68071; WB: 1:10 000); goat anti-mouse Alexa Fluor594 (Thermo Fisher Scientific; product number A11005; immunofluorescence: 1:500).

### Antibody production

RNJ1 is a tau-specific antibody that was isolated following the panning of the Tomlinson I and J scFv library with recombinant full-length hTau. To generate the RNJ1 IgG, the variable heavy and variable light sequences were cloned into murine IgG1 and kappa mAbXpress vectors.^22^ Large-scale low-endotoxin recombinant antibody production was conducted (Queensland node of the National Biologics Facility) and the final preparation was stored in 1× phosphate-buffered saline at −80°C. The hTau-specific antibody, HJ8.5, was a gift from Professor Jürgen Götz at the University of Queensland and was generated as previously described.^1^ A mammalian expression plasmid encoding the RNJ1 scFv was generated by cloning the variable light and variable heavy domains, joined by a (Gly4Ser)3 linker and in frame with a C-terminal triple Flag tag, into pcDNA™6.2/V5-DEST (Thermo Fisher Scientific).

### Preparation of recombinant tau protein

Full-length hTau (hTau441) was cloned into the pET-DEST42 vector (Thermo Fisher Scientific) in frame with C-terminal V5 and His6 tags. Plasmids were transformed into One Shot® BL21™ bacterial cells (Thermo Fisher Scientific), and recombinant protein expression was induced with 1 mM isopropyl 1-thio-β-d-galactopyranoside for 2 h at 37°C. The bacterial suspension was pelleted at 4000 × *g* for 15 min at 4°C and then resuspended in immobilized metal affinity chromatography buffer (300 mM KCl, 50 mm KH_2_PO_4_ and 5 mM imidazole, pH 8.0) containing 0.1% Complete protease inhibitor and 0.1-mg/ml lysozyme (Merck Millipore) followed by incubation on ice for 20 min. The cells were then subjected to repeated freeze/thawing after which they were sonicated at a 60% amplitude for 1 min. The lysate was centrifuged at 16 000 × *g* for 20 min at 4°C and filtered through a 0.22-μm syringe filter (Merck Millipore). To purify hTau441, the supernatant was passed over a 1-ml Bio-Scale Mini Profinity immobilized metal affinity chromatography cartridge (Bio-Rad) and then eluted in 300 mM KCl, 50 mM KH_2_PO_4_ and 250 mM imidazole, pH 8.0. Eluted proteins underwent buffer exchange into 137 mM NaCl, 2.7 mM KCl, 4.3 mm Na_2_HPO_4_ and 88 mM KH_2_PO_4_, pH 7.4 and were then subjected to size exclusion chromatography using an S200 10/30 GL column (GE Healthcare) equilibrated in 1× phosphate-buffered saline. Fractions corresponding to the protein of interest were combined and concentrated using an Amicon Ultra Filter with a 3000-kDa molecular mass cut-off (Merck Millipore). Full-length mouse tau was prepared as previously described.^23^

### Enrichment of insoluble tau from human brain tissue

Post-mortem brain tissue samples were obtained from the Victorian Brain Bank (Australia) and met the standard criteria for Alzheimer’s disease neuropathological diagnosis. Biochemical extraction of the insoluble tau was achieved as previously described.^24^ Briefly, 1 g of grey matter from post-mortem frontal cortex tissues was homogenized in 10 volumes (*v*/*w*) of lysis buffer (10 mM Tris–HCl, pH 7.4, 800 mM NaCl, 1 mM ethylenediaminetetraacetic acid, 2 mM dithiothreitol and 10% sucrose) using a probe sonicator (Branson, Sonifier® Cell Disruptor) and then centrifuged at 16 000 × *g* for 20 min at 4°C (Eppendorf micro-centrifuge, 5415D). The crude supernatant was treated with N-lauroylsarcosinate [1% (*w*/*v*) final concentration] and shaken at room temperature for 1 h. The supernatant was then centrifuged at 100 000 × *g* for 1 h at 4°C. The supernatant was collected as ‘sarkosyl soluble’. The sarkosyl-insoluble pellet was resuspended in 300-μl washing buffer (10 mM Tris–HCl, pH 7.4, 800 mM NaCl, 5 mM ethylenediaminetetraacetic acid, 2 mM dithiothreitol and 10% sucrose) and centrifuged at 16 000 × *g* for 30 min at 4°C. Following centrifugation, the supernatant was further centrifuged at 100 000 × *g* (Beckman Coulter, Optima MAX-XP) for 1 h at 4°C. Finally, the purified sarkosyl-insoluble pellet was resuspended in 50-μl 20 mM Tris–HCl, pH 7.4, and 100 mM NaCl and stored at −80°C. Total protein concentration was measured using a BCA protein assay kit (Thermo Fisher Scientific). All procedures were approved by the University of Melbourne human research ethics committee and in accordance with the Australian National Health and Medical Research Council guidelines.

### Characterization of RNJ1 binding by immunoblotting

Soluble and insoluble tau fractions (5 μg) were electrophoresed on a 10% Tris–glycine SDS–PAGE gel. Proteins were transferred to low fluorescence polyvinylidene fluoride membrane (Thermo Fisher Scientific) using the iBlot 2 System (Thermo Fisher Scientific); then, membranes were washed and blocked for 1 h in Odyssey® Blocking Buffer (LI-COR). Membranes were incubated with RNJ1 mAb overnight at 4°C by rocking. Membranes were washed with TBS-T and incubated with the secondary antibody (LI-COR) for 1 h at room temperature. Membranes were imaged using the Odyssey Fc Imaging System (LI-COR). Image processing was performed on Image Studios software (LI-COR).

### Enzyme-linked immunosorbent assay

The binding specificity of RNJ1 mAb was determined using an enzyme-linked immunosorbent assay as previously described.^1^ Briefly, Immuno 96 MicroWell plates (Nunc) were coated with 10-μg/ml recombinant mouse tau or hTau tau, followed by incubation with phosphate-buffered saline, or either RNJ1 mAb or controls Tau-5 or HJ8.5 at 1 μg/ml. Plates were blocked in 3% bovine serum albumin and bound antibodies were detected with anti-mouse horseradish peroxidase conjugate (1:5000), followed by incubation with the substrate solution 3,3′,5,5′-tetramethylbenzidine (Merck Millipore). The absorbance was measured at 450 nm using a POLARstar OPTIMA plate reader (BMG Labtech).

### mRNA production

Synthetic genes (gBlocks) encoding the RNJ1 IgG HC, LC and scFv with 5′ T7 promoter and 3′ and 5′ untranslated regions (UTRs) were generated (Integrated DNA Technologies). The scFv gene was in frame with a C-terminal Flag tag for detection. The gBlocks were prepared following the manufacturer’s instructions and polymerase chain reaction amplified prior to IVT. The polymerase chain reaction products were purified using the QIAquick polymerase chain reaction Purification Kit (QIAGEN) and 0.5 µg underwent IVT using the HiScribe T7 Anti-Reverse Cap Analog mRNA Kit with tailing (New England Biolabs) according to the manufacturer’s instructions. The mRNA was purified using the Monarch RNA Clean-up Kit (New England Biolabs). A yield of ∼30 µg of mRNA was produced for each preparation.

### Cell culture

Wild-type SH-SY5Y cells and SH-SY5Y transfected with full-length hTau with a C-terminal GFP tag (Tau-GFP cells) were cultured in Dulbecco’s modified Eagle’s media (Gibco), supplemented with 10% (*v*/*v*) foetal calf serum (Gibco) and 1% (*v*/*v*) penicillin–streptomycin (Gibco) at 37°C with 5% CO_2_. Tau-GFP cells were selected with 6-mg/ml blasticidin (Thermo Fisher Scientific). For expression of the RNJ1 IgG, wild-type SH-SY5Y cells were maintained in 90% (*v*/*v*) CD Hybridoma Media (Gibco), 10% (*v*/*v*) Dulbecco’s modified Eagle’s media (Gibco) and supplemented with 1% (*v*/*v*) foetal calf serum and 0.1% (*v*/*v*) penicillin–streptomycin.

### Cell transfections

Prior to transfection, SH-SY5Y cells were plated in culture media at a density of 2 × 10^5^ cells per well of a 12-well plate to achieve 80–90% confluency at the time of transfection and incubated for 24 h at 37°C. For mRNA, transfection was performed using Lipofectamine MessengerMax (Thermo Fisher Scientific) according to the manufacturer’s protocol. For transfection of the IgG HC and LC mRNA, 1.5–3 μg of mRNA was used, whereas 0.15–1.2 μg of mRNA was used for transfection of the scFv mRNA. RNJ1 scFv plasmid DNA (pDNA) transfection was conducted with 1.6-μg pDNA using Lipofectamine 2000 (Thermo Fisher Scientific) according to the manufacturer’s protocol. To determine antibody expression, the cells and media were harvested 24–72 h after transfection.

### Immunoblotting to determine protein expression following mRNA delivery

Medium collected from the SH-SY5Y cells transfected with mRNA encoding the IgG HC and the IgG LC and co-transfected with both was diluted in 4× NuPage LDS Sample Buffer (Thermo Fisher Scientific). For reduced conditions, 10× NuPage Reducing Agent (Thermo Fisher Scientific) was added and samples were heated at 70°C for 10 min. Samples were electrophoresed and western blotted with the anti-mouse secondary antibody only. To determine the expression of the RNJ1 intrabody, SH-SY5Y cells transfected with the mRNA encoding the RNJ1 scFv were lysed in 1× RIPA buffer (Abcam), and total protein concentration was measured using a BCA protein assay kit (Thermo Fisher Scientific). Three- to five-microgram total protein was diluted in 4× NuPage LDS Sample Buffer (Thermo Fisher Scientific) with 10× NuPage Reducing Agent (Thermo Fisher Scientific) and heated at 70°C for 10 min. Samples were electrophoresed and western blotted with anti-Flag antibody (Cell Signalling Technology). All membranes were imaged using the Odyssey Fc Imaging System (LI-COR). Image processing was performed on Image Studios software (LI-COR).

### Immunofluorescence and microscopy

Tau-GFP cells plated onto glass coverslips were transfected with 1.6-μg RNJ1 scFv pDNA using Lipofectamine 2000 or 0.6-μg RNJ1 scFv mRNA with Lipofectamine MessengerMax as per manufacturer recommendations for 24 h. The next day, cells were fixed and immunolabelled with anti-Flag antibody followed by goat anti-mouse Alexa Fluor594. Cell nuclei were labelled using 4′,6-diamidino-2-phenylindole (Sigma-Aldrich) (immunofluorescence: 1:1000). Cells were imaged with the Crest Spinning Disk (Nikon) using a 60× oil objective. The system was controlled by NIS Elements acquisition software. Confocal *z*-stacks were imported into ImageJ, and single *z*-slices with respective orthogonal views were captured for image presentation. Co-localization analysis was conducted on the entire *z*-stack for single cells using the co-localization plugin, Coloc2, calculating Pearson’s correlation coefficient. Pearson’s coefficient (*r*) measures the degree to which signal intensities in two channels are linearly correlated to each other, by which *r* = 1 depicts perfect positive correlation and *r* = 0 depicts no correlation. Confocal *z*-stacks were also imported into IMARIS analysis software, and transfection efficiency was determined using the Spots creation tool to quantify transfected (Flag-positive) and total cells. Estimated XY diameter for Spot detection was 5 μm. Mouse hippocampal primary neurons (8 days in vitro) plated out onto glass coverslips were transfected with 0.5-µg mRNA encoding the RNJ1 scFv using Lipofectamine MessengerMax. Eight days later, cells were fixed and underwent immunofluorescent labelling with anti-Flag to detect RNJ1 scFv and Tau-5 to detect tau. The cell nucleus was labelled using 4′,6-diamidino-2-phenylindole. Cells were imaged with the Axio Observer 7 inverted microscope (Zeiss) using a 40× oil objective. System was controlled using the ZEN acquisition software. For further processing, images were *z*-projected by the averaging method in ImageJ processing software.

### Immunoblotting to determine IgG binding to tau

The hTau441 (5 μg) was electrophoresed and immunoblotted with either Tau-5, RNJ1 monoclonal antibody or the undiluted media collected from SH-SY5Y cells co-transfected with mRNA encoding the RNJ1 IgG HC and LC. Membranes were imaged as described above.

### Tau pull-down assay

Tau-GFP cells were plated and transfected with RNJ1 scFv mRNA as described above. Twenty-four hours after transfection, cells were collected and lysed in immunoprecipitation buffer [20 mM Tris–HCl pH 7.4, 10 mM potassium chloride, 10 mM magnesium chloride, 2 mM EDTA, 10% (*v*/*v*) glycerol and 1% (*v*/*v*) Triton X-100]. The lysate (‘Input’) was incubated with anti-GFP antibody overnight at 4°C with rotation. Following overnight incubation, 50-µL protein-G agarose beads (Roche) were then added to each sample and incubated for 1 h at room temperature with rotation. Samples were centrifuged at 5000 × *g* for 2 min, and supernatant (‘flow-through’) was collected for western blotting. The pulled-down sample was washed thrice with lysis buffer and pulled down protein eluted (‘immunoprecipitation’) by boiling in 1× NuPAGE LDS Sample Buffer (Thermo Fisher Scientific). Samples were electrophoresed and western blotted with Tau-5 antibody and anti-Flag antibody.

### Statistical analysis

Statistical analyses were performed using GraphPad Prism 9.0 software. Normal data distribution was analysed using the Shapiro–Wilk test. Statistical significance between experimental groups was analysed using either one-way or two-way ANOVA followed by Tukey’s multiple comparisons test or unpaired *t*-test, where *P* ≤ 0.05 was considered statistically significant. All values are reported as mean ± SD of the mean.

## Results

### mRNA encoding RNJ1 IgG and scFv is synthesized with a 5′ cap and 3′ poly(A) tail

**Figure 1:**
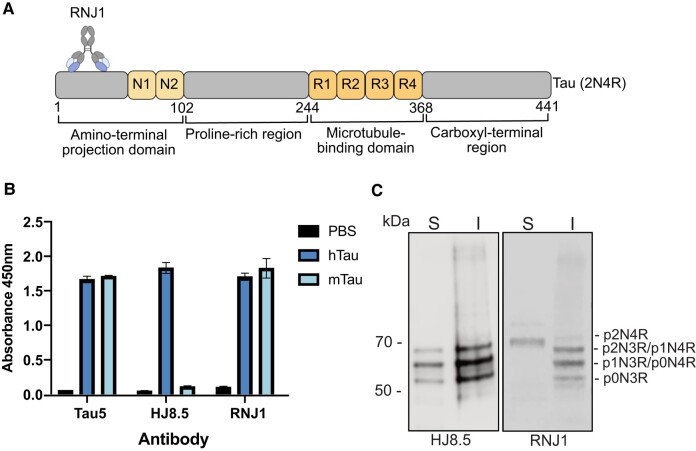
**Characterization of RNJ1 antibody.** (**A**) Schematic representation of tau-specific antibody, RNJ1, bound to tau at amino acids 1–22 of full-length hTau (2N4R). (**B**) Enzyme-linked immunosorbent assay of the RNJ1 antibody in comparison with phosphate-buffered saline, the commercially available pan-tau antibody, Tau-5 and the hTau-specific antibody, HJ8.5, confirming that RNJ1 binds both mouse and hTau (mean ± SD, *n* = 3). (**C**) WB analysis of sarkosyl-soluble (S) and sarkosyl-insoluble (I) cortical brain homogenates derived from Alzheimer’s disease brain probed with tau-specific antibodies RNJ1 (pan tau) and HJ8.5 (pan tau). Schematic created with BioRender.

RNJ1 is a tau-specific antibody that binds within amino acids 1–22 of full-length tau (Fig. 1A).^25^ RNJ1 detects both mouse and hTau, similar to the commercially available tau antibody, Tau-5 and unlike HJ8.5, a hTau-specific antibody that has been explored in human clinical trials (Fig. 1B). Further comparison of RNJ1 and HJ8.5 specificity to insoluble tau isolated from human post-mortem brain homogenate reveals RNJ1 is capable of binding to tau species found in tau paired helical filaments (Fig. 1C; Supplementary Fig. 1).

**Figure 2:**
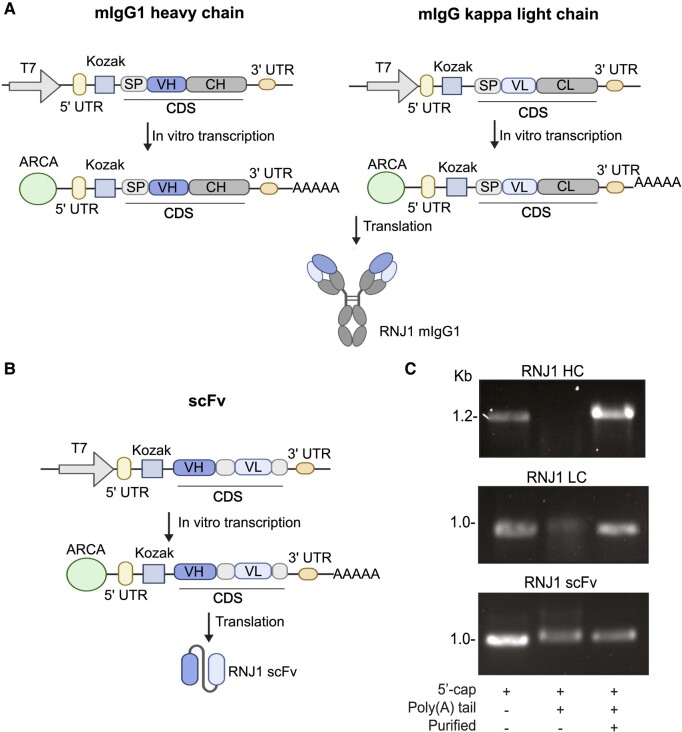
**IVT synthesis of RNJ1 IgG and scFv mRNA.** (**A**) Schematic of RNJ1 murine IgG HC and kappa LC DNA templates and IVT mRNA. The murine IgG HC and LC pair combine to form the RNJ1 IgG (**B**) scFv DNA template and the subsequent IVT mRNA. (**A**, **B**) Each DNA template is composed of a T7 promoter, a 5′ and 3′ UTR a Kozak sequence and the coding sequence. The IgG HC coding sequence consists of a signal peptide, the RNJ1 variable heavy domain and a mouse IgG1 constant heavy domain. The IgG LC coding sequence consists of a signal peptide, the RNJ1 variable light domain and a mouse IgG kappa constant light domain. The RNJ1 scFv coding sequence consists of the RNJ1 variable heavy and variable light domains joined by a flexible glycine–serine linker. The final IVT-synthesized mRNA contains a 5′ Anti-Reverse Cap Analog cap and a poly(A) tail (AAAAA). (**C**) Agarose gel loaded of IVT mRNA after 5′ capping, addition of the poly(A) tail and purification (kb, kilobase). Schematics created with BioRender. ARCA, Anti-Reverse Cap Analog; CDS, coding sequence; CH, constant heavy; CL, constant light; SP, signal peptide; VH, variable heavy; VL, variable light.

To synthesize the IVT mRNA of RNJ1, synthetic double-stranded DNA encoding the RNJ1 murine IgG1 HC, murine kappa LC and RNJ1 scFv was generated. Each synthetic DNA construct contained the bacteriophage T7 promoter at the 5′ end followed by a 5′ UTR, a Kozak sequence, a transcriptional start site and a 3′ UTR (Fig. 2A and B). The commonly used secretory peptide from interleukin-2 was used for the secretion of the expressed RNJ1 IgG HC and LC.^26^ Following IVT, the mRNA contained the synthetic Anti-Reverse Cap Analog, which prevents capping of mRNA in reverse orientation, thereby ensuring all transcripts are translatable. In addition to the 5′ Anti-Reverse Cap Analog cap, all mRNA constructs were synthesized to include a 3′ poly(A) tail, estimated to be 150 nucleotides or longer (Fig. 2A and B). Gel electrophoresis confirmed successful IVT synthesis of mRNA (Fig. 2C; Supplementary Fig. 2).

### Co-transfection of IgG HC and LC mRNA results in the expression and secretion of functional full-length RNJ1 IgG

**Figure 3:**
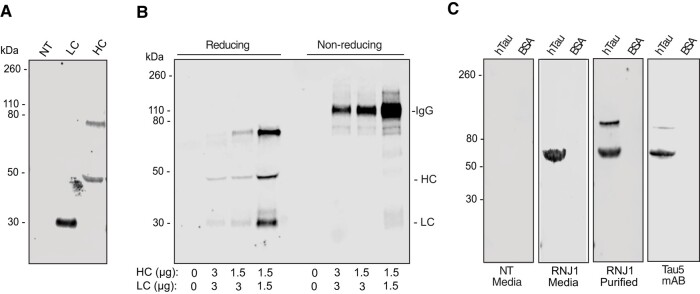
**RNJ1 IgG expression and secretion following mRNA transfection.** (**A**) WB analysis of media collected from non-transfected SH-SY5Y cells or SH-SY5Y cells transfected with IgG HC or IgG LC mRNA. Samples were electrophoresed under reducing conditions and probed with an anti-mouse secondary antibody. (**B**) WB of media collected from non-transfected SH-SY5Y cells (0 μg of HC and LC) or SH-SY5Y cells transfected with either 1.5 or 3 μg of mRNA at different ratios of IgG HC to IgG LC (1:1 or 1:2). Samples were electrophoresed under reducing or non-reducing conditions and probed with anti-mouse secondary antibody. (**C**) WB analysis of recombinant hTau probed with media collected from non-transfected SH-SY5Y cells (non-transfected media) or SH-SY5Y cells co-transfected with RNJ1 IgG HC and LC mRNA (RNJ1 media), compared with immunoblots probed with recombinant RNJ1 (RNJ1 purified) or Tau-5 (Tau-5 purified). Bovine serum albumin was used a negative control for binding. All primary antibodies were detected with an anti-mouse secondary antibody. BSA, bovine serum albumin; NT, non-transfected.

To determine if the delivery of mRNA encoding an IgG can result in the production and secretion of a functional antibody in human SH-SY5Y neuroblastoma cells, the mRNA encoding the HC and LC of RNJ1 was individually transfected or co-transfected into SH-SY5Y cells and the media was analysed. Following individual transfections, both the RNJ1 HC and LC were translated and secreted into the media (Fig. 3A; Supplementary Fig. 3A). Western blotting revealed expression of the HC at the expected size of 50 kDa (Fig. 3A) and the LC at the expected size of 25 kDa (Fig. 3A; Supplementary Fig. 3A). A HC dimer of 90 kDa was also observed (Fig. 3A; Supplementary Fig. 3A).

The successful generation of full-length IgG protein via co-transfection of IVT mRNA encoding the HC and LC has previously been reported as challenging due to several methodological variables, including finding the optimal mRNA LC to HC ratio, and appropriate delivery method of the mRNA (e.g. parallel, subsequent or simultaneous delivery).^27^ We therefore tested two different HC to LC mRNA ratios (1:1 and 1:2) using parallel co-transfection. Both conditions showed comparable generation and secretion of RNJ1 IgG at the expected size of 195 kDa under non-reducing conditions (Fig. 3B; Supplementary Fig. 3B). The correct formation of the RNJ1 IgG was further confirmed under reducing conditions when the RNJ1 IgG disulphide bonds were broken to display the separated HC (50 kDa) and LC (25 kDa) (Fig. 3B; Supplementary Fig. 3B). As observed in the individual transfections, the HC formed a dimer of ∼90 kDa in size (Fig. 3B; Supplementary Fig. 3B). Interestingly, an increased amount of HC and LC mRNA (3 μg of each, 1:1) showed reduced HC and LC expression and lower RNJ1 IgG levels (Fig. 3B; Supplementary Fig. 3B). A reduced amount of 1.5 μg of mRNA at a 1:1 ratio of HC to LC was, therefore, used for subsequent transfections.

Once expression of the RNJ1 murine IgG1 was achieved, the ability of the mRNA-encoded IgG to bind tau was assessed by immunoblotting electrophoresed human recombinant tau with media collected from SH-SY5Y cells transfected with RNJ1 HC and LC. Similar to the positive control antibodies, Tau-5 and recombinant purified RNJ1 antibody, immunoblotting with the media from the SH-SY5Y cells co-transfected with RNJ1 HC and LC resulted in a positive signal at 70 kDa, corresponding to human recombinant tau (Fig. 3C; Supplementary Fig. 3C). A higher molecular weight band at ∼100 kDa was detected with Tau-5 and the recombinant purified RNJ1 antibody, but not with the RNJ1 IgG in the media (Fig. 3C; Supplementary Fig. 3C). This band may correspond to an aggregated tau species and the inability of mRNA-encoded RNJ1 to detect this minor species may be due to a reduced concentration of RNJ1 IgG secreted into the media compared with the highly concentrated purified RNJ1 antibody.

### Transfection of SH-SY5Y cells with mRNA encoding RNJ1 scFv results in intrabody expression

**Figure 4:**
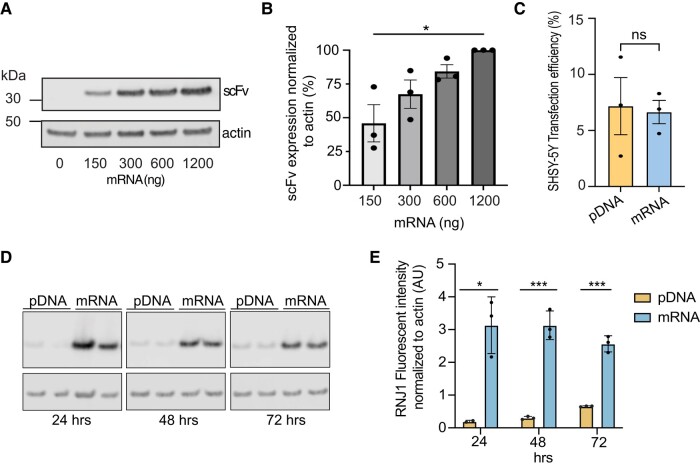
**Expression of mRNA-encoded RNJ1 scFv intrabody.** (**A**) Representative WB of cell lysates from non-transfected SH-SY5Y cells (0-ng mRNA) or SH-SY5Y cells transfected with an increasing amount of RNJ1 scFv mRNA (150–1200 ng). Immunoblots were probed with an anti-Flag antibody to detect the RNJ1 scFv and an anti-β-actin antibody as a loading control. (**B**) Quantification of RNJ1 scFv fluorescent intensity, normalized to β-actin fluorescence and plotted as a percentage (%) of the highest mRNA dose (mean ± SD; *n* = 3), one-way ANOVA with Tukey’s multiple comparison test (* = *P* < 0.05). (**C**) Quantification of calculated transfection efficiency (%) from SH-SY5Y cells transfected with 0.8-pmol RNJ1 scFv mRNA or 0.8-pmol RNJ1 scFv pDNA for 24 h (mean ± SD; *n* = 3), unpaired *t*-test. (**D**) Representative WB of cell lysates from SH-SY5Y cells transfected with either RNJ1 scFv mRNA or RNJ1 scFv pDNA for 24, 48 and 72 h. Immunoblots were probed with an anti-Flag antibody to detect the RNJ1 scFv and an anti-β-actin antibody as a loading control. (**E**) Quantification of RNJ1 scFv fluorescent intensity in **D**, normalized to β-actin expression (mean ± SD; *n* = 3), two-way ANOVA with Tukey’s multiple comparison test (*P < 0.05, **P < 0.01 and ***P < 0.001).

Tau is predominately localized within neurons where it aggregates and induces neurotoxicity in disease. Tau antibody therapeutics may, therefore, have enhanced efficacy if they are able to engage intracellular tau and prevent tau aggregation. To investigate whether transfection of cells with mRNA encoding the RNJ1 scFv results in the expression of a functional tau intrabody, SH-SY5Y cells were transfected with various amounts of IVT mRNA encoding the RNJ1 scFv. Analysis of the mRNA-transfected cell lysates revealed the successful generation of the RNJ1 scFv with an increase in intrabody expression levels with an increasing amount of mRNA used for transfection (Fig. 4A and B; Supplementary Fig. 4A). The mRNA transfection efficiency and protein translation in SH-SY5Y cells was then compared with the conventional pDNA to determine mRNA validity. Comparison of the SH-SY5Y transfection efficiency with equal molar concentrations of RNJ1 scFv mRNA and pDNA demonstrated a similar transfection efficiency (6.64% for mRNA and 7.17% for pDNA) between the two nucleic acid species, suggesting mRNA does not compromise transfection efficiency in SH-SY5Y cells (Fig. 4C). Intrabody expression following transfection with equal amounts of mRNA and pDNA was, therefore, examined at 24, 48 and 72 h after transfection, and interestingly, an increase in RNJ1 scFv protein expression was observed at all time points following mRNA transfection compared with pDNA transfection, suggesting mRNA transfection may result in higher protein expression levels (Fig. 4D and E; Supplementary Fig. 4B). Protein levels did not differ significantly over time within each transfection group (Fig. 4D and E).

### RNJ1 intrabody engages intracellular tau

**Figure 5:**
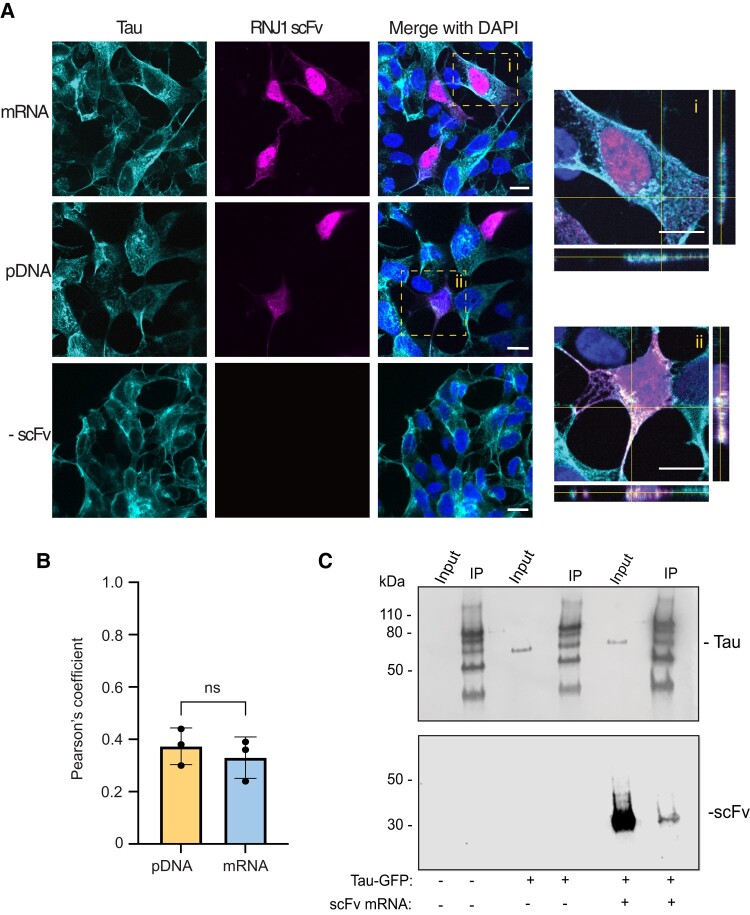
**RNJ1 intrabody binds intracellular tau.** (**A**) Representative immunofluorescent imaging (60×) of fixed tau-GFP SH-SY5Y cells either non-transfected (-scFv) or transfected with RNJ1 scFv mRNA or RNJ1 scFv pDNA. hTau was detected by its GFP fusion, and an anti-Flag antibody was used to detect the RNJ1 scFv. The nuclear marker, 4′,6-diamidino-2-phenylindole (DAPI), was used to identify all cells. (i and ii) Representative *Z*-stack images (60×) with orthogonal views showing overlap of RNJ1 scFv fluorescence and tau fluorescence in the cell cytoplasm (white) following (i) RNJ1 scFv mRNA transfection or (ii) RNJ1 scFv pDNA transfection. Scale bars represent 10 µm. (**B**) Pearson’s correlation coefficient between tau and RNJ1 scFv delivered as pDNA or mRNA. (**C**) Representative WB of the cell lysates from wild-type SH-SY5Y cells or Tau-GFP SH-SY5Y cells non-transfected or transfected with RNJ1 scFv mRNA, before (input) and after GFP-specific immunoprecipitation. Immunoblot was probed with an anti-Flag antibody to detect the RNJ1 scFv or Tau-5 to detect Tau. IP, immunoprecipitation.

Having confirmed RNJI intrabody expression, localization and binding to intracellular tau were, therefore, investigated. Following transfection of murine primary hippocampal neurons with mRNA encoding the RNJ1 scFv, the RNJ1 intrabody was shown to be localized within the soma and neuronal processes of the neurons, similar to its target, tau (Supplementary Fig. 5A). Co-localization analysis of RNJ1 intrabody with tau was, therefore, investigated. Immunofluorescent imaging of Tau-GFP SH-SY5Y cells transfected with mRNA encoding the RNJ1 scFv revealed a surprising predominant localization of the intrabody in the nucleus and, to a lesser extent, in the cytoplasm (Fig. 5A). This differed to that of the RNJ1 scFv pDNA transfected cells, which revealed a more evenly distributed localization of RNJ1 throughout the cell (Fig. 5A), suggesting mRNA could affect the cellular localization of the translated protein. Orthogonal sections of the cytoplasm showed the RNJ1 intrabody and tau both localized in the *z*-plane (Fig. 5Ai and ii). Furthermore, a Pearson’s coefficient was calculated to be 0.37 for pDNA transfected cells and 0.33 for mRNA transfected cells, suggesting close proximity of the RNJ1 intrabody and tau.

To confirm an interaction between tau and the RNJ1 intrabody, a GFP-pull-down assay was conducted following the transfection of Tau-GFP SH-SY5Y cells with RNJ1 scFv mRNA. Tau and RNJ1 were detected in the pull-down demonstrating positive engagement between the mRNA-encoded RNJ1 intrabody and intracellular tau (Fig. 5B; Supplementary Fig. 5B).

## Discussion

The clinical development of therapeutic antibodies is hindered by the high costs associated with large-scale production and frequent dosing regimens that are required to observe disease-modifying effects. Strategies to enhance antibody bioavailability within the brain will likely improve therapeutic outcomes and reduce costs associated with the treatment of neurodegenerative diseases like Alzheimer’s disease. IVT mRNA encoding antibody therapeutics presents a promising alternative to conventional passive immunotherapy and overcomes the need to generate recombinant antibodies. Here, we demonstrate that IVT-synthesized mRNA encoding a therapeutic tau antibody, RNJ1, in an IgG and scFv format, results in the endogenous translation of the antibodies when delivered to human neuroblastoma cells. Furthermore, we show that IVT mRNA encoding the RNJ1 scFv co-localizes with tau in the cytoplasm. Importantly, the endogenously translated RNJ1 in both the full-sized IgG and smaller scFv format can engage its target, tau. For the translated RNJ1 scFv, tau engagement was shown to occur within the cell cytoplasm, making it the first documented evidence, to our knowledge, of a direct interaction between a tau antibody and tau within the cell.

Whilst targeting intracellular tau, rather than extracellular tau, may be a better therapeutic strategy for the treatment of Alzheimer’s disease, full-sized IgGs are ideal for targeting extracellular proteins such as the amyloid-β peptide that accumulates to form amyloid plaques in Alzheimer’s disease. The protocol developed in this study for the effective production of IVT mRNA encoding a functional tau-targeting IgG could be applied to the development of next-generation amyloid-β-specific monoclonal antibodies such as lecanemab. A recent study by Wu *et al*.^28^ compared the serum concentration of a bi-specific antibody targeting PD-L1 and PD-1 for the treatment of intestinal cancer, delivered as IVT mRNA or a recombinant protein, and found that a single injection of the bi-specific antibody mRNA, encapsulated within lipid nanoparticles, resulted in more robust serum antibody levels with enhanced duration compared the recombinant bi-specific antibody from mammalian cell culture sources.^28^ Therefore, from a pharmacokinetic perspective, the IVT mRNA technology may be advantageous compared with the delivery of recombinant antibodies.

It is important to highlight that the IVT mRNA in this study was generated from synthetic double-stranded DNA templates, rather than linearized plasmids. This strategy overcomes the need for time-consuming and expensive plasmid-based cloning and facilitates the rapid construction of mRNA sequence variants designed for enhanced translation, stability and functionality.^29^ Potential limitations of synthetic mRNA therapeutics are their immunogenicity and propensity to be rapidly degraded. Comparison of mRNA transcripts with high GC content to those with low GC content has demonstrated, however, that increased GC content can improve protein expression by helping synthetic mRNA evade targeted degradation by adenylate-/uridylate-rich element-binding proteins and endoribonucleases and minimize immunogenicity of the synthetic mRNA by decreasing interaction with Toll-like receptors that may trigger innate immune activation.^30-33^ Furthermore, a high GC content has been demonstrated to be more optimally translated compared with AU-rich transcripts.^34^ Sequence optimization of the UTRs of the mRNA transcript can also improve translation. The 5′ UTR is a critical region required for ribosomal binding and initiation of translation, and optimization of the 5′ UTR sequence has been demonstrated to increase protein translation efficiency.^35-37^ The 3′ UTR is another critical region, important for mRNA transport to specific cellular compartments.^38^ Surprisingly, we observed the RNJ1 scFv intrabody predominantly localized in the nucleus when delivered as mRNA. As nuclear RNJ1 was observed to a lesser extent following pDNA delivery, it is plausible that the 3′ UTR of the RNJ1 mRNA transcript affected its localization within the cell. Future sequence optimization of the RNJ1 scFv 3′ UTR could reduce intrabody localization in the nucleus and increase intrabody localization in the cytoplasm to improve tau engagement.

Antibody transcripts, specifically, can be optimized to enhance antibody translation and stability. Antibody secretion may be improved using an alternative signal peptide. A study by Rybakova *et al*.^27^ compared multiple signal peptides to enhance serum levels of a therapeutic anti-HER2 monoclonal antibody, trastuzumab, following IVT mRNA delivery *in vivo*.^27^ Their study revealed that although intracellular levels of trastuzumab were not affected by the signal peptide sequence, the mRNA containing human Ig kappa LC signal peptide resulted in higher levels of trastuzumab in the cell medium compared with trastuzumab with GLuc and H5/L1 signal peptides.^27^ Intrabody mRNA, on the other hand, can be optimized for enhanced therapeutic purposes by increasing the stability of the scFv sequence. Cytoplasmic expression of antibodies is often challenging due to the reducing environment of the cell cytosol and the propensity of antibodies to form insoluble aggregates in this environment.^39^ It is, therefore, possible that the localization of the mRNA-delivered RNJ1 intrabody in the nucleus improved intrabody stability and contributed to its enhanced protein levels when compared with the RNJ1 intrabody expressed from pDNA. Several groups have demonstrated that intrabody stability and expression can be improved by reducing the overall charge of the translated protein.^39,40^ This can be achieved through the fusion of highly charged peptide tags to the antibody transcript. Furthermore, the functionalization of intrabodies can be achieved by adding protein degradation moieties to the scFv sequence for enhanced clearance of the target protein.^16,41^

Finally, *in vivo* mRNA immunotherapy studies have been limited to targeting peripheral targets^27,28,42,43^ as mRNA is predominantly encapsulated in lipid nanoparticles that are unable to effectively enter the brain. Delivery to the central nervous system would require packaging of the mRNA into vehicles capable of crossing the blood–brain barrier. To date, mRNA delivery to the brain following intravenous administration has not been achieved, and therefore, *in vivo* studies have utilized intracranial injection of the mRNA into the brain.^44^ Unfortunately, this is a significant hurdle of the translation of mRNA neurotherapeutics to the clinic and future work should be directed at optimizing mRNA delivery across the blood–brain barrier. Furthermore, our study has focused on the tau antibody, RNJ1, a pan-tau-specific antibody unable to distinguish pathological tau species from normal functioning tau. In a therapeutic context, RNJ1’s hypothesized mechanism of action would, therefore, be to prevent pathological tau–tau interactions, rather than clear pathogenic tau. Importantly, however, IVT mRNA technology is not limited to RNJ1 and can be applied to the delivery of any other tau antibody that has therapeutic potential.

In conclusion, here, we show that IVT-synthesized mRNA can be used to generate both functional full-length IgG antibodies and scFv intrabodies targeting tau. Our study demonstrates the utility of mRNA as a delivery platform for antibody therapeutics.

## Supplementary Material

fcae100_Supplementary_Data

## Data Availability

The data that support the findings of this study are available from the corresponding author, upon reasonable request.
