# Supplementary material for: Messenger RNA-encoded antibody approach for targeting extracellular and intracellular tau
Source: Brain Commun. 2024 Mar 25;6(2):fcae100. doi: 10.1093/braincomms/fcae100 (PMC10996922; doi:10.1093/braincomms/fcae100)
Supplement: fcae100_Supplementary_Data [file fcae100_supplementary_data.pdf]

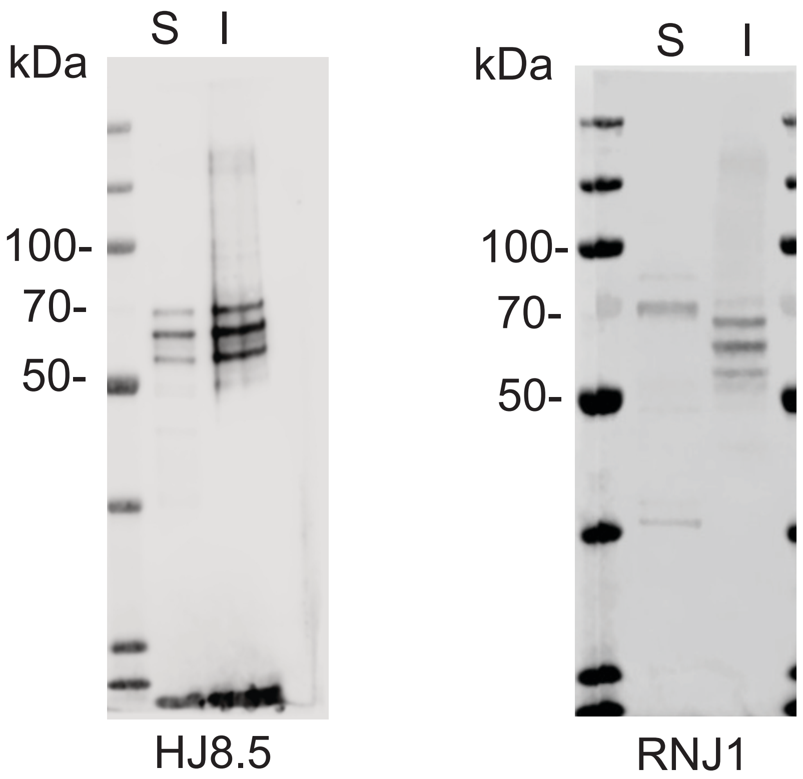

Supplementary Figure 1. Uncropped western blot images of Fig. 1C corresponding to analysis of sarkosyl-soluble (S) and -insoluble (I) cortical brain homogenates derived from Alzheimer's disease brains probed with tau-specific antibodies RNJ1 (pan tau) and HJ8.5 (pan tau).

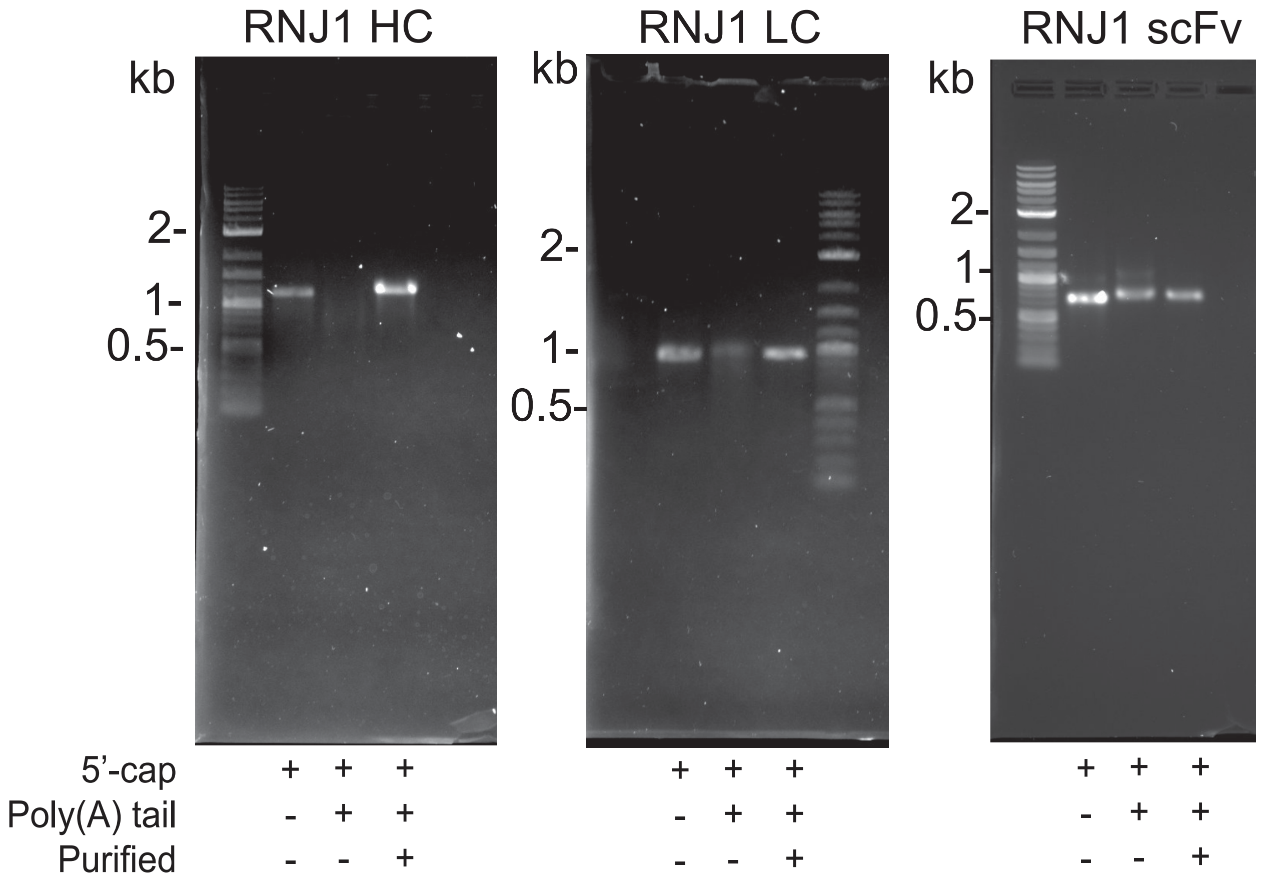

Supplementary Figure 2. Uncropped agarose gel images from Fig. 2C corresponding to IVT mRNA after 5' capping, addition of the poly(A) tail, and purification.

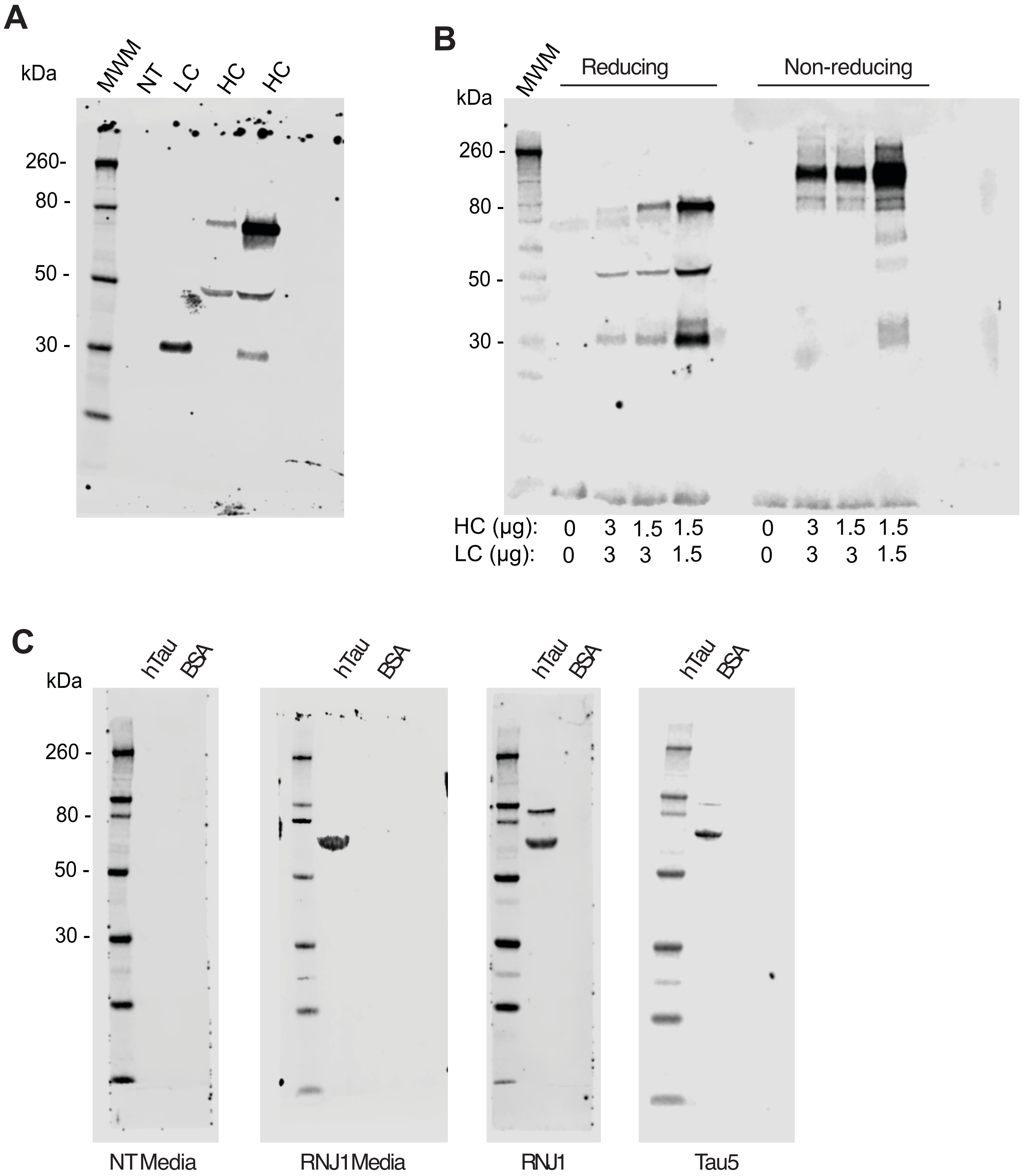

Supplementary Figure 3. (A) Uncropped western blot images of Fig. 3A corresponding to analysis of media from SH-SY5Y cells non-transfected or transfected with IgG heavy chain (HC) and/or IgG light chain (LC) mRNA and probed with anti-mouse secondary antibody. (B) Uncropped images from Fig. 3B corresponding to a western blot of media collected from SH-SY5Y cells non-transfected or transfected with either 1.5 or 3µg of HC and LC at different ratios (1:1 or 1:2 HC:LC) (C) Uncropped images of Fig. 3C corresponding to western blots of recombinant human tau probed with media collected from non-transfected or RNJ1 IgG HC and LC mRNA-transfected SH-SY5Y cells, compared to immunoblots probed with recombinant RNJ1 or Tau5.

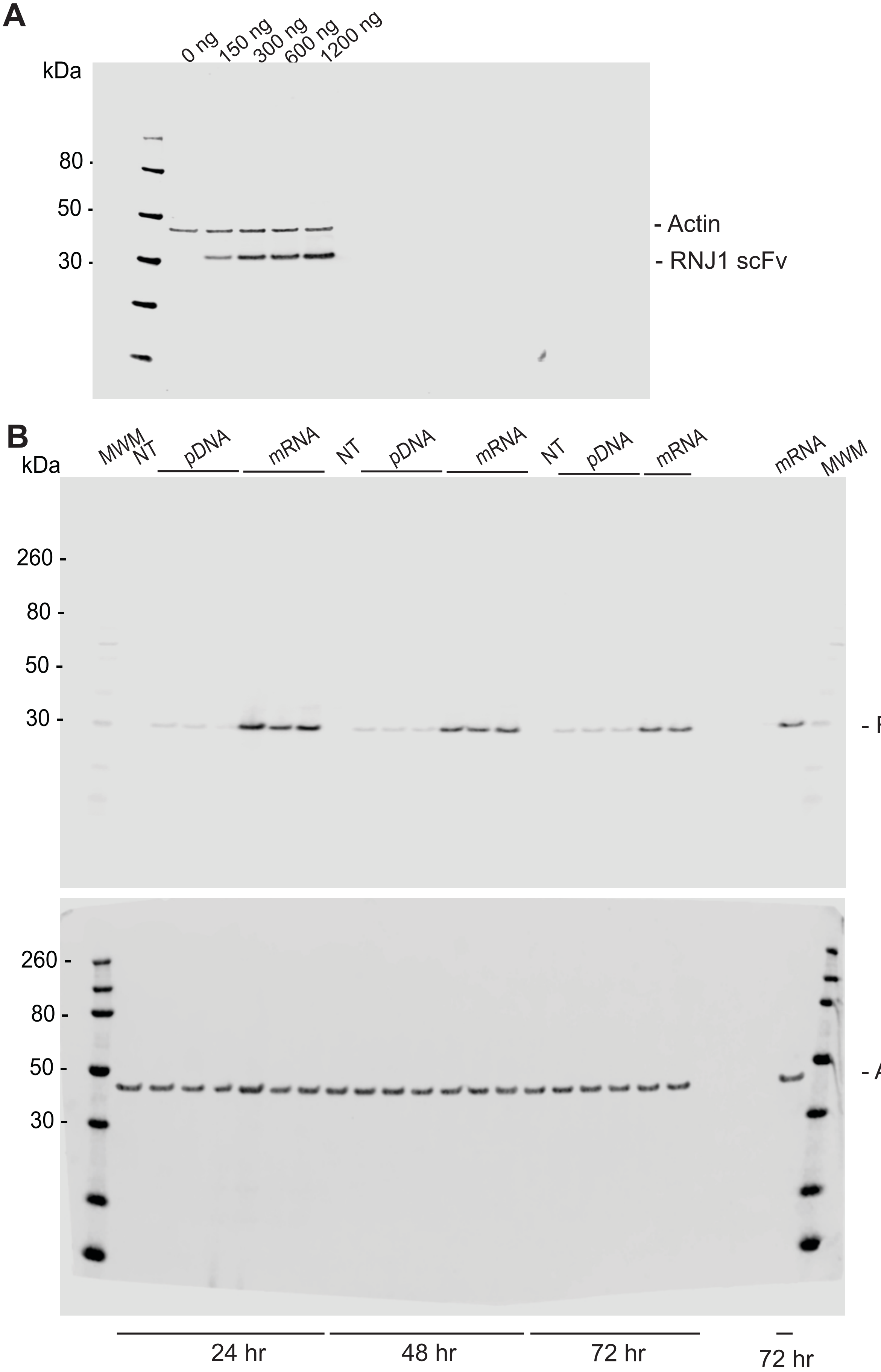

Supplementary Figure 4. (A) uncropped image of Fig. 4A corresponding to a western blot of cell lysates from non-transfected SH-SY5Y cells (0 ng) or SH-SY5Y cells transfected with an increasing amount of RNJ1 scFv mRNA (150-1200 ng). Immunoblots were probed with an anti-FLAG antibody to detect the RNJ1 scFv and an anti--actin antibody as a loading control. (B) uncropped image of Fig. 4D corresponding to a western blot of cell lysates from SH-SY5Y cells transfected with either RNJ1 scFv mRNA or RNJ1 scFv plasmid DNA (pDNA) for 24, 48, and 72 hours. Immunoblots were probed with an anti-FLAG antibody to detect the RNJ1 scFv and an anti-actin antibody as a loading control.

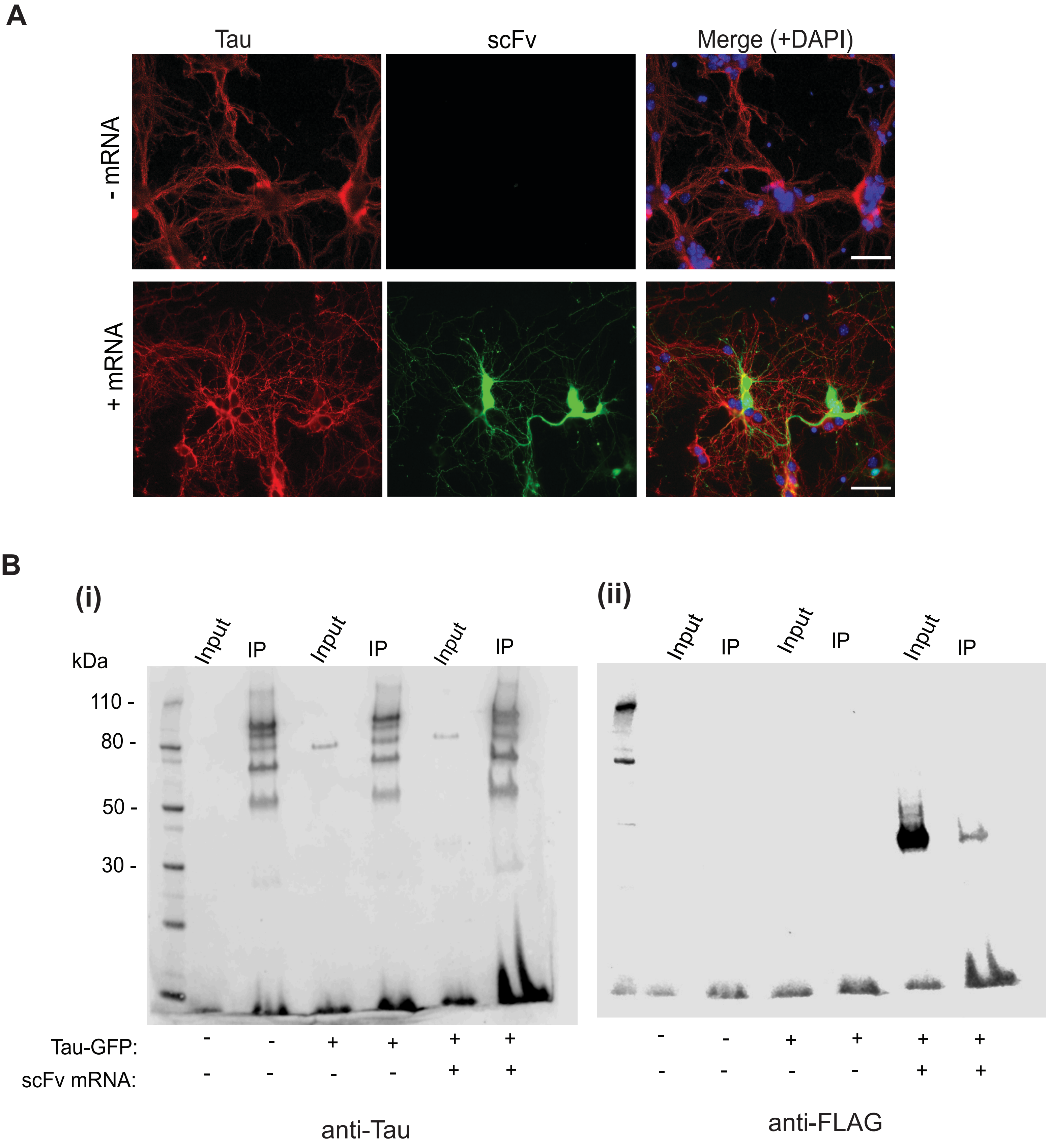

Supplementary Figure 5. (A) Neurons were probed for intracellular Tau with Tau-5 (red), RNJ1 with α-Flag (green), and DAPI (4′,6-diamidino-2-phenylindole) (blue) as a nuclear marker. (B) Uncropped images of Fig. 5C corresponding to western blots of the cell lysates from wild-type SH-SY5Y cells, or Tau-GFP SH-SY5Y cells non-transfected or transfected with RNJ1 scFv mRNA, before (input) and after GFP-specific immunoprecipitation (IP). Immunoblot was probed with Tau-5 to detect Tau (i) or anti-FLAG antibody to detect the RNJ1 scFv (ii).
